# Another brick in the wall

**DOI:** 10.1590/S1516-31802002000200001

**Published:** 2002-03-02

The history of lung cancer spans many years, and over recent decades excision of tumors has been considered as the only effective treatment. In 1933 Graham performed the first successful pneumonectomy due to lung cancer. In 1937 Ochsner and DeBakey first concluded that smoking is a cause of lung cancer.^1^ This important observation was confirmed by Doll and Hill who firmly established the relationship between smoking and lung cancer in 1950.^2^ The Sixties are considered to be the decade when modern chemotherapy and radiotherapy came into being. Despite all the progress, the cure rate for lung cancer has only increased from 9% to 14%.^3^ However, the introduction of new technologies such as molecular biology and genetics has opened up significant prospects for improvement through the use of biomarkers and risk/susceptibility factors.

Epidemiological evidence suggests that lung cancer may show familial aggregation, after adjusting for cigarette smoking and other risk factors, and that susceptibility to lung cancer may be inherited in a Mendelian fashion. There is evidence that lung cancer and other smoking-related cancers have an inherited genetic component. However, the existence of such a genetic component has yet to be proven. The first piece of evidence to support the possibility of an inherited component was the familial aggregation or clustering of such diseases. In 1963 Tokuhata and Lilienfeld observed, in a case-control study, that the number of deaths caused by lung cancer was four times higher among relatives of lung cancer patients than among relatives of non-cigarette smoking controls and two times higher among cigarette-smoking relatives.^4^

Since then, several other studies have demonstrated a familial component to lung cancer risk (including an article of relevance in this issue of the *São Paulo Medical Journal*^5^). However, the consistent demonstration of lung cancer aggregation in families may allow other interpretations. Differences in family structure (family size and ages) between cases and controls or environmental risk factors such as cigarette smoking shared among family members may affect the risk profile of the family.

Lung cancer is among the top three leading types of cancer, and it is the leading cause of cancer death, with the death rate mirroring its prevalence. For every new case there is a death, meaning that minimal progress has been made in early detection and treatment. The identification of at-risk populations will allow resources to be concentrated on early detection, with an improvement in outcomes. The well-structured article by Wünch-Filho et al.^5^ in this issue adds new evidence to the concept of genetic risk factors for lung cancer.
